# Novel Single Inhibitor of HDAC6/8 and Dual Inhibitor of PI3K/HDAC6 as Potential Alternative Treatments for Prostate Cancer

**DOI:** 10.3390/ph14050387

**Published:** 2021-04-21

**Authors:** Fabiana Sélos Guerra, Daniel Alencar Rodrigues, Carlos Alberto Manssour Fraga, Patricia Dias Fernandes

**Affiliations:** 1 Laboratório de Farmacologia da Dor e da Inflamação, Instituto de Ciências Biomédicas, Universidade Federal do Rio de Janeiro, Rio de Janeiro 21941-901, Brazil; fabianasellos@hotmail.com; 2 Programa de Pós-graduação em Farmacologia e Química Medicinal, Instituto de Ciências Biomédicas, Universidade Federal do Rio de Janeiro, Rio de Janeiro 21941-901, Brazil; 3 Laboratório de Avaliação e Síntese de Substâncias Bioativas (LASSBio), Instituto de Ciências Biomédicas, Universidade Federal do Rio de Janeiro, Rio de Janeiro 21941-901, Brazil; dar.go.qfm@gmail.com (D.A.R.); cmfraga@ccsdecania.ufrj.br (C.A.M.F.)

**Keywords:** cancer, prostate cancer, PI3K, HDAC6, apoptosis, cell cycle

## Abstract

Background: Prostate cancer is the second most frequently diagnosed malignancy worldwide. Here, the cytotoxic and antimetastatic effects of a new HDAC6/8 inhibitor, LASSBio-1911, and a new dual-PI3K/HDAC6 inhibitor, LASSBio-2208, were evaluated against PC3 prostate cancer cell line. Methods: A MTT assay was used to assess the cell viability. Annexin V/propidium iodide (PI) was used to detect apoptotic cell death and to analyze the cell cycle distribution. Interleukin 6 (IL-6) levels were measured by ELISA. A cell scratch assay was performed to assess cell migration, and the expression of proteins was estimated by Western blotting. Results: LASSBio-1911 and LASSBio-2208 exert cytotoxic effects against PC3 cells. However, LASSBio-2208 was demonstrated to be more potent than LASSBio-1911. The apoptosis assays showed that both compounds trigger apoptotic processes and cause the arrest of cells in the G2/M phase of the cell cycle. The Western blot analysis revealed that LASSBio-2208 significantly decreased the expression of p-JNK and JAK2. However, both compounds reduced the expression of p-STAT3, IL-6 secretion, and cell migration. Conclusions: LASSBio-1911 and LASSBio-2208 demonstrated significant activity in reducing cell viability and migration. These compounds can be further used as prototypes for the development of new potential anticancer alternative treatments.

## 1. Introduction

Prostate cancer is the second most frequently diagnosed malignancy (after lung cancer) in men worldwide and tends to develop in older men aged 50 years and older [1]. Prostate tumors are often cured when they are still localized; however, the formation of metastases can occur in up to 25% of patients [2]. The vast majority of patients diagnosed with metastatic disease remain asymptomatic for months to years [3], which makes diagnosis and treatment more difficult. The disease is initially sensitive to androgen deprivation therapies (the castration-sensitive disease state), but resistance is inevitably acquired, leading to an incurable state [2]. Thus, there is an urgent need for new therapeutic agents capable of inhibiting the progression and metastasis of cancer cells during early treatment.

Phosphoinositide 3-kinase (PI3K) signaling has emerged as an attractive target for cancer therapy, and many drugs that inhibit various PI3K pathway components are currently in clinical trials. PI3K is a heterodimeric protein that participates in multiple cellular processes, including cell proliferation, transformation, migration, and differentiation [4]. Therefore, inhibitors of PI3K are considered to be candidate drugs for cancer therapy; however, the results from clinical trials with PI3K inhibitors and solid tumors have been largely disappointing because of several factors, such as drug-related toxicities, feedback upregulation of compensatory mechanisms when PI3K is blocked, and few studies using combinations with other antagonists exists so far [5].

Histone deacetylase (HDAC) modulates epigenetic and nonepigenetic mechanisms such as cell cycle arrest and apoptosis and other forms of cancer cell death [6]. Therefore, overexpression of HDAC6 is related to tumor cell invasion and metastasis [7]. HDAC inhibitors have shown promising results against haematological malignancies, but depending on the cancer type and genetic background, the response to HDAC inhibitors may depend on a specific biological response [8].

Fu and colleagues [9] showed that the dual blockade of PI3K/HDAC potently inhibited the proliferation of pancreatic cancer cell lines, and Seidel and colleagues [10] showed that an HDAC6 inhibitor exhibited antitumor effects alone or in combination with other drugs, such as PI3K inhibitors, in various cancers. The dual inhibition of PI3K/HDAC6 is a promising strategy for the treatment of certain types of intractable cancers because of its advantages in overcoming potential resistance and producing synergistic effects. Thus, the development of a dual PI3k/HDAC inhibitor is a reasonably attractive strategy [11].

In this study, we investigated LASSBio-2208 and LASSBio-1911 against PC3 prostate cancer cells.

## 2. Results

### 2.1. LASSBio-1911 and LASSBio-2208 Reduced the Viability of PC3 Prostate Cancer Cells

**Figure 1:**
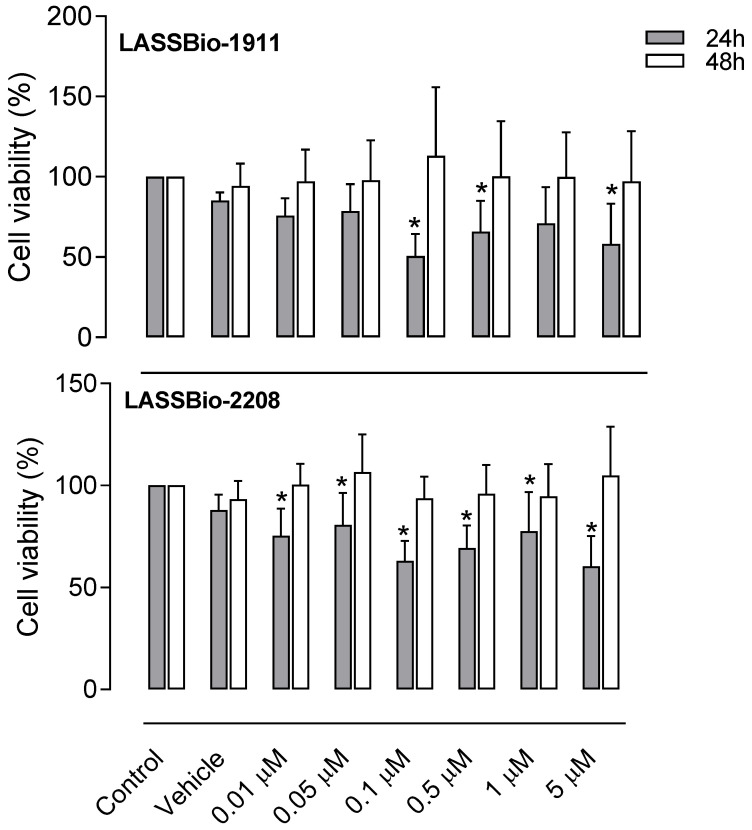
LASSBio-1911 and LASSBio-2208 inhibited the viability of human PC3 cells. An MTT assay was used to examine the viability of human PC3 cells. The cells were plated in 96-well plates and exposed to several concentrations of LASSBio-1911 and LASSBio-2208 for 24 and 48 h. All the values are presented as the mean ± standard error of three independent experiments. Statistical analysis was performed using GraphPad Prism 8.02 and one-way ANOVA with Dunnett’s post-test, and statistical significance was defined as * *p* < 0.05 compared with untreated cells (controls).

To examine the cytotoxic effects of LASSBio-1911 and LASSBio-2208 on prostate cancer PC3 cells, an MTT assay was conducted. After 24 h of treatment with 0.1, 0.5, and 5 μM LASSBio-1911 and all concentrations of LASSBio-2208, the viability of PC3 cells was reduced compared with that of untreated cells. However, after 48 h of treatment, no reduction in cell viability was observed following incubation with either compound (Figure 1).

### 2.2. LASSBio-1911 and LASSBio-2208 Induced the Apoptosis of PC3 Cells

**Figure 2:**
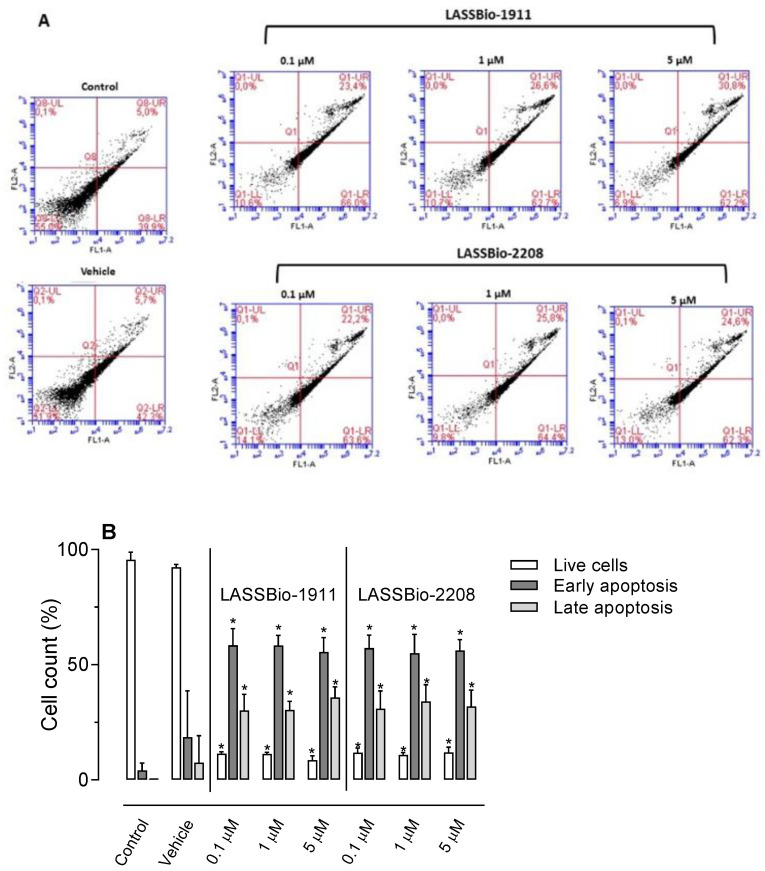
LASSBio-1911 and LASSBio-2208 triggered the apoptosis of PC3 cells. Cells were treated with LASSBio-1911 and LASSBio-2208 for 24 h. (**A**) shows a representative analyses of flow cytometry (FACS) after double-staining cells with Annexin V-FITC and PI, and (**B**) presents the percentage of live cells in early or late apoptosis. All the values are presented as the mean ± standard error of three independent experiments. Statistical analysis was performed using GraphPad Prism 8.02 and one-way ANOVA followed by Dunnett’s post-test, and statistical significance was defined as * *p* < 0.05 compared with untreated cells (controls).

To understand the mechanisms involved in cell death, we carried out Annexin V/PI double staining followed by flow cytometry analysis to detect early and late apoptotic PC3 cells after LASSBio-1911 and LASSBio-2208 treatment. At all the concentrations tested, both compounds caused 60–66% early apoptosis and up to 20–30% late apoptosis within 24 h of treatment (Figure 2A,B). In contrast, nontreated cells showed normal cell viability without significant changes in death rate.

**Figure 3:**
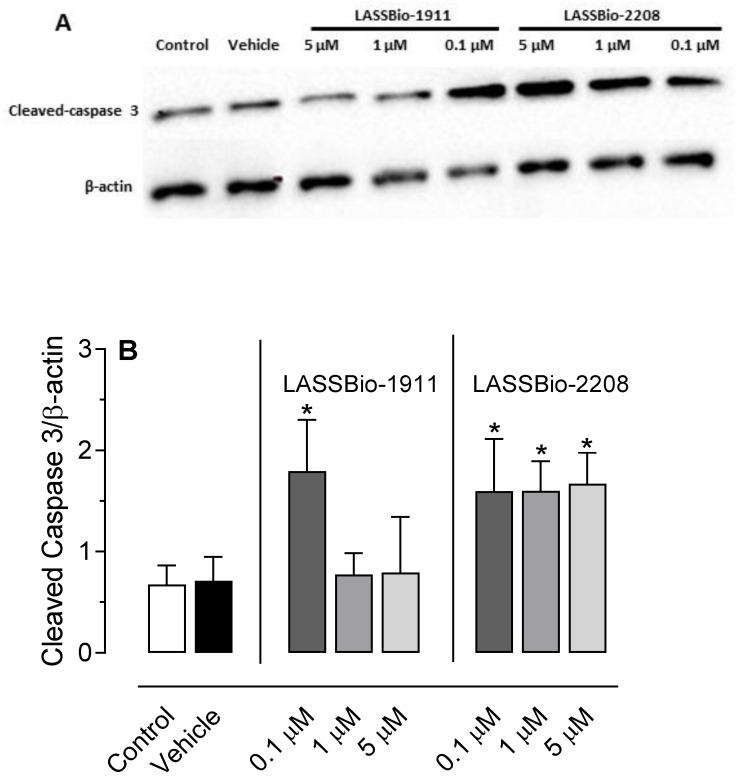
LASSBio-1911 and LASSBio-2208 triggered the apoptosis of PC3 cells. PC3 cells were treated with LASSBio-1911 or LASSBio-2208 (0.1, 1 or 5 µM) for 24 h. (**A**) presents a representative Western blot showing cleaved caspase 3 and β-actin (loading control). (**B**) shows the arbitrary units for the cleaved caspase 3/actin ratio. All the values are presented as the mean ± standard error of three independent experiments. Statistical analysis was performed using GraphPad Prism 8.02 and one-way ANOVA followed by Dunnett´s post-test, and statistical significance was defined as * *p* < 0.05 compared with untreated cells (controls).

We next evaluated the effect of LASSBio-1911 and LASSBio-2208 against the expression of cleaved caspase 3 through Western blot analysis. The data shown in Figure 3 reveal that treatment with LASSBio-1911 (at 0.1 µM) and LASSBio-2208 (at all concentrations) significantly increased the activity of caspase after 24 h (Figure 3A,B). These results suggest that both compounds induced the apoptosis of PC3 cells.

### 2.3. Effects of LASSBio-1911 and LASSBio-2208 on Cell Cycle Distribution

To further evaluate whether both compounds affect cell cycle phases, we studied the effects of LASSBio-1911 and LASSBio-2208 on the cell cycle progression of PC3 cells. Table 1 shows cell arrest in G2/M phase after 24 h of treatment with either LASSBio-1911 or LASSBio-2208.

Cell samples were stained with propidium iodide, and the cellular DNA content was determined by flow cytometry. All the values are presented as the mean ± standard error of three independent experiments. Statistical analysis was performed using GraphPad Prism 8.02 and one-way ANOVA followed by Dunnett´s post-test, and statistical significance was defined as * *p* < 0.05 compared with untreated cells (controls).

### 2.4. LASSBio-1911 and LASSBio-2208 Inhibited PC3 Cell Proliferation via the JNK Pathway

**Figure 4:**
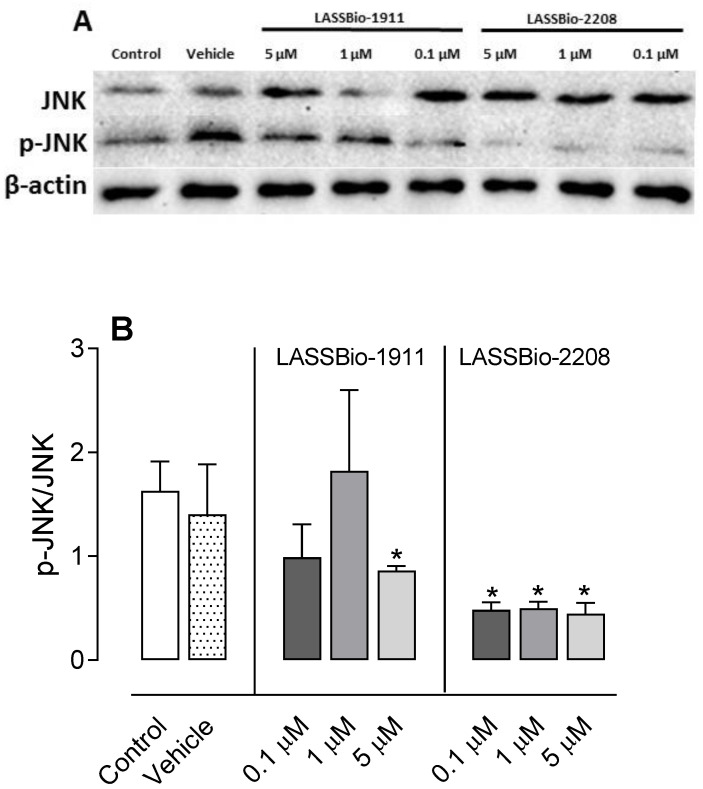
Effects of LASSBio-1911 and LASSBio-2208 on the phosphorylation of JNK in PC3 cells. Cells were treated with compounds (0.1, 1 and 5 μM) for 24 h. (**A**) show a representative Western blot of p-JNK, JNK and β-actin proteins. (**B**) shows the p-JNK/JNK ratio values. The data are presented as the mean ± standard error of three independent experiments. Statistical analysis was performed using GraphPad Prism 8.02 and one-way ANOVA followed by Dunnett´s post hoc test. Statistical significance was defined as * *p* < 0.05 compared with untreated cells (controls).

To understand the molecular mechanism by which LASSBio-1911 and LASSBio-2208 induce PC3 apoptosis, we analyzed the changes in the activation of various signaling pathways like JNK-signaling pathway that is required for growth of prostate carcinoma cells in vitro and in vivo. JNK exerts functions to control a wide variety of cell processes including cell apoptosis, proliferation, migration, survival, differentiation, and inflammation through activating a number of nuclear and non-nuclear molecules [12,13,14]. LASSBio-2208 treatment markedly reduced JNK phosphorylation (Figure 4), and the effect was much more pronounced than that of LASSBio-1911 treatment. According to these results, the dual inhibition of PI3K/HDAC6 leads to a more effective inhibition of the JNK pathway, which may contribute to the activation of the apoptotic process in PC3 cells.

### 2.5. LASSBio-2208 Inhibited JAK/STAT3 Phosphorylation in PC3 Cells

**Figure 5:**
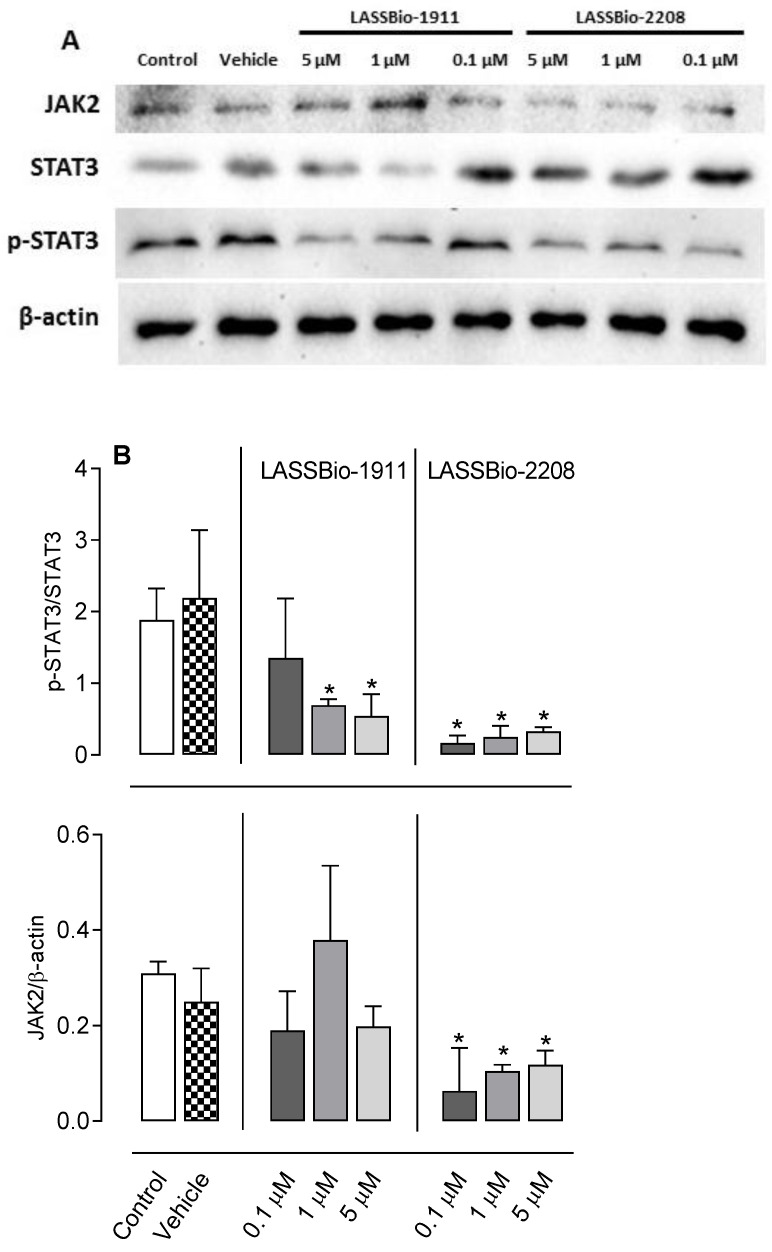
LASSBio-1911 and LASSBio-2208 reduce STAT3 phosphorylation and JAK2 expression in prostate cancer cells. (**A**) presents a representative Western blot showing the expression of STAT3, p-STAT3, and JAK2 enzymes. (**B**) shows the values of the p-STAT3/STAT3 and JNK/β-actin ratios. The data are presented as the mean ± standard error of three independent experiments. Statistical analysis was performed using GraphPad Prism 8.02 and one-way ANOVA followed by Dunnett´s post hoc test. Statistical significance was defined as * *p* < 0.05 compared with untreated cells (controls).

We performed Western blotting to analyze the expression of JAK2/STAT3 signaling pathway components. As shown in Figure 5, after 24 h of treatment, both compounds inhibited STAT3 phosphorylation in PC3 cells. LASSBio-1911 significantly inhibited STAT3 expression at concentrations of 1 and 5 µM, and LASSBio-2208 demonstrated an important effect, reducing STAT3 expression by almost 90% at all concentrations used. On the other hand, only the dual inhibitor LASSBio-2208 significantly reduced JAK2 expression after incubation at all three concentrations (0.1, 1 and 5 µM).

### 2.6. LASSBio-1911 and LASSBio-2208 Inhibited IL-6 Secretion from PC3 Cells

**Figure 6:**
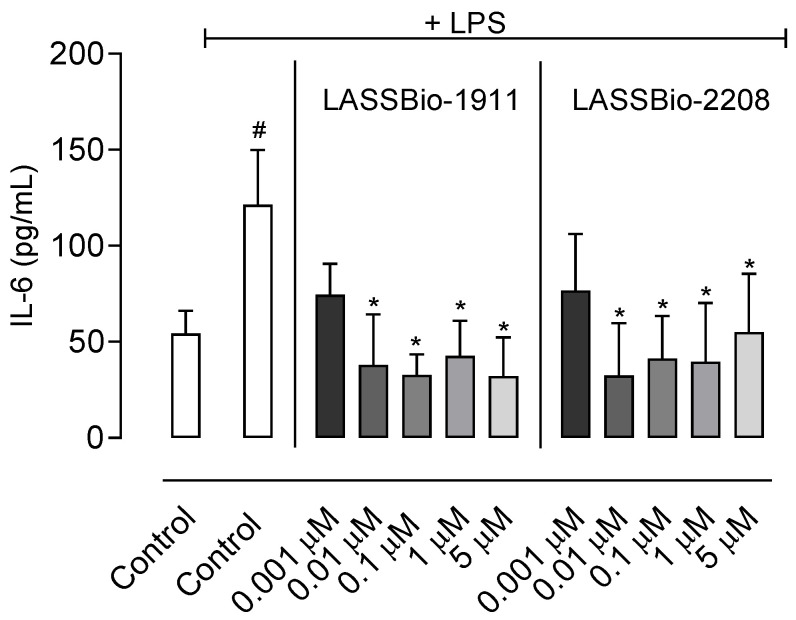
The effects of LASSBio-1911 and LASSBio-2208 on IL-6 production in PC3 cells. Cells were treated with LASSBio-1911 or LASSBio-2208 (0.001–5 μM) for 24 h. The results are expressed as the mean ± standard error of five independent experiments. Statistical analysis was performed using GraphPad Prism 8.02 and one-way ANOVA with Dunnett´s post-test, and statistical significance was defined as * *p* < 0.05 compared with the LPS-treated group. ^#^ *p* < 0.05 compared with the non-LPS treated group (control).

Previous studies have indicated that IL-6 drives many of the expression of cancer ‘hallmarks’ through the downstream activation of the JAK/STAT3 signaling pathway [15,16]. Therefore, to further examine whether this cytokine participates in LASSBio-1911 and LASSBio-2208 interference of cell migration, PC3 cells were cultured for 24 h in the presence or absence of LASSBio-1911 or LASSBio-2208 (0.001–5 µM). To stimulate IL-6 cell secretion, LPS (1 µg/mL) was added after 30 min of treatment. The data obtained showed that both compounds, LASSBio-1911 and LASSBio-2208, significantly inhibited the production of cytokine IL-6 at concentrations between 0.01 and 5 µM (Figure 6).

### 2.7. Effects of LASSBio-1911 and LASSBio-2208 on the Motility of Prostate Cancer Cells

**Figure 7:**
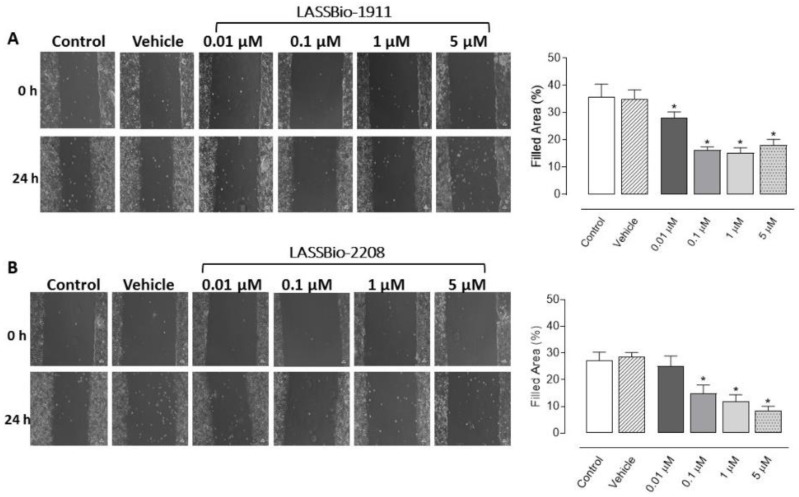
LASSBio-1911 (**A**) and LASSBio-2208 (**B**) reduce cell migratory capacity. PC3 cell monolayers were scratched with a pipette tip and incubated with LASSBio-1911 or LASSBio-2208 for 24 h. The images on the left are representative photomicrographs of wound healing obtained at a magnification of 10× (scale bar = 50 µM). The graphs show the number of cells migrating into the scratched zone. All the values are presented as the mean ± standard error of three independent experiments. Statistical analysis was performed using GraphPad Prism 8.02 and one-way ANOVA with Dunnett´s post hoc test, and statistical significance was defined as * *p* < 0.05 compared with untreated cells (controls).

To analyze whether LASSBio-1911 and LASSBio-2208 can alter cell migration, we performed a cell scratch assay followed by quantification of the wound area. The results demonstrated that fewer treated cells than control cells migrated in the wounded area when the cells were exposed to 0.1, 1, or 5 µM LASSBio-1911 (Figure 7A) or LASSBio-2208 (Figure 7B) for 24 h. The quantitative data revealed that both compounds significantly suppressed the motility of PC3 cells. These results indicate that the inhibition of HDAC6/8 and the dual inhibition of PI3K/HDAC6 lead to a reduction in STAT3 phosphorylation, which leads to a decrease in IL6 secretion and, as a consequence, reduced autocrine stimulation of IL-6 receptors, culminating in the inhibited migration of the PC3 cells.

## 3. Discussion

In a previous study, we demonstrated that LASSBio-1911 and LASSBio-2208 can inhibit cell viability and migration by inhibiting tubulin assembly in human prostate tumor cells, DU145, but not in human prostate normal cells, RWPE-1 [17]. In this study, we evaluated the effects of LASSBio-1911 and LASSBio-2208 on PC3 prostate tumor cells and our results showed an important action of the compounds in inhibiting the proliferation and migration of these tumor cells. The MTT assay was used to detect the effects of these compounds on cell viability. We found that both compounds were able to reduce cell viability after 24 h of incubation (Figure 1), but even though LASSBio-1911 was able to affect cell viability, higher doses were required than needed for LASSBio-2208 to have the same effect. Previous studies [3,18] showed an important reduction in the cell viability of different types of tumors upon the inhibition of HDAC6; however, other studies have shown that single PI3K inhibition is rarely cytotoxic and is more commonly cytostatic [19,20], because upon inhibition of the PI3K pathway, cells enter a dormant, nutrient-deprived state, but do not necessarily die. Thus, after 48 h of incubation with both compounds, we did not observe a reduction in cell viability and this event may be related to the induction of cell cycle arrest and a greater mitochondrial activity of cells during the apoptotic process [21,22,23]. These results corroborate our findings that the dual inhibition of PI3K and HDAC6 through LASSBio-2208 may be a better alternative for reducing the viability of prostate tumor cells, as it was more potent than the inhibition of only HDAC6/8. Additionally, in our recent study, we showed that LASSBio-2208 treatment caused a much less potent cytotoxic effect on RWPE-1 cells than LASSBio-1911, indicating that the dual inhibitor LASSBio-2208 has a much more selective cytotoxic profile for tumor cells [17].

Even with the results showing that both compounds LASSBio-1911 and LASSBio-2208 reduced cell viability by activating the apoptotic pathway, we further examined the effects of both compounds on the cell cycle. Targeting the aberrant expression of critical proteins involved in cell cycle progression has become a novel strategy for cancer intervention [24]. Our results demonstrated that both the single inhibitor of HDAC 6/8 (LASSBio-1911) and the dual inhibitor of PI3K/HDAC6 (LASSBio-2208) caused G2/M cell cycle arrest of PC3 cells, indicating that HDAC6 is essential for this effect. These results are consistent with earlier reports with other PI3K/HDAC inhibitors [25,26,27].

We also demonstrated that LASSBio-1911 and LASSBio-2208 inhibited cellular proliferation by inducing apoptosis through activation of caspase 3. These results are partially consistent with published data from Lee and collaborators [28] that demonstrated that a combination of a selective HDAC6 inhibitor and ibrutinib (a tyrosine kinase inhibitor) was effective in reducing cell viability and upregulating cellular apoptosis in non-Hodgkin lymphoma and follicular lymphoma. Another study showed that the dual inhibition of PI3K/HDAC by 24 h of CUDC-907 treatment can activate the apoptosis pathway in thyroid cancer cell lines by significantly increasing the activity of caspase 3/7 [29].

The JNK signaling pathway has an important role in regulating tumor cell apoptosis [30]. Zhao and colleagues [31] showed that both the PI3K/Akt and JNK pathways are essential for glioblastoma cell survival, migration and invasion and that the inhibition of PI3K and JNK exhibited synergistic effects on suppressing glioblastoma cell proliferation and migration. Thus, we evaluated whether LASSBio-1911 and LASSBio-2208 modulate the JNK pathway. We have shown that LASSBio-2208 significantly decreased the expression of p-JNK and increased the expression of JNK, but LASSBio-1911 was unable to affect the expression of p-JNK, indicating an important relationship between PI3K inhibition and the reduced activation of the JNK pathway. Our results corroborate those of a previous study that revealed that the PI3K/AKT/JNK signaling pathways are important for inducing the apoptosis of ovarian cancer cells [32].

The JAK/STAT signaling axis plays a major role in the proliferation and survival of different cancer cells [33]. A previous study demonstrated that enhancing the JAK/STAT signaling pathway can facilitate prostate cancer pathogenesis due to the processes of cell proliferation, invasion and apoptosis are affected [34]. Therefore, we questioned whether the dual inhibition of PI3K/HDAC6 or the single inhibition of HDAC6/8 is involved in the activation of the JAK/STAT3 pathway in PC3 cells. Our data showed that only the dual inhibitor LASSBio-2208 reduced the expression of JAK2 and p-STAT3, and the HADC6/8 inhibitor LASSBio-1911 demonstrated an effect only in reducing the expression of p-STAT3. Activation of STAT3 signaling is essential for the metastatic progression of prostate cancer, and targeting the STAT3 pathway can be a potential therapeutic intervention for prostate cancer [35]. Thus, our results demonstrate that the inhibition of HDAC6 by LASSBio-1911 and LASSBio-2208 is essential to reduce the phosphorylation and activation of STAT3 in PC3 cells, which indicates that these compounds may be less likely to develop resistance mechanisms such as that observed for vorinostat [36]. However, other studies still need to be performed to confirm this hypothesis.

IL-6 is a potent inflammatory cytokine and IL-6 can activate signal transduction through the PI3K signaling pathway [37]. Chang and colleagues [38] demonstrated that the IL-6/JAK/STAT3 signaling pathway drives tumorigenesis and metastasis. The activation of Toll-like receptors (TLRs) leads to a significant increase in IL-6 secretion by bone marrow-derived macrophages [39]. Pei and colleagues [40] demonstrated that PC3 human prostate epithelial cells constitutively express TLR4, which plays an important role in cancer development. PC3 cells respond to LPS stimulation through TLR4 signaling. Our data suggest that both compounds can reduce the secretion of IL-6 after LPS stimulation, corroborating results of previous studies that showed the involvement of the IL-6/JAK/STAT3 signaling pathway in the progression of prostate cancer and that the inhibition of p-STAT3 is essential to reduce IL-6 release.

We analyzed the migration of PC3 cells based on the following considerations: IL-6 may promote the initial steps of prostate cancer metastasis by upregulating MMP-9 through the PI3K/Akt signaling pathway [41], the inhibition of JAK/STAT3 activity or the interception of microtubule assembly to suppress diffuse large B-cell lymphoma migration [42]; the downregulation of STAT3 can reverse the inhibited invasion and migration of oesophageal squamous cell carcinoma [43]; and our results indicated that LASSBio-1911 and LASSBio-2208 inhibit the IL-6/STAT3 signaling pathway. The data obtained showed that the inhibition of HDAC6/8 or the dual inhibition of PI3K/HDAC6 led to a reduction in the migration of PC3 cells, suggesting that both compounds have antimetastatic activity through the inhibition of the IL-6/STAT3 pathway.

## 4. Materials and Methods

### 4.1. LASSBio-1911 and LASSBio-2208 Synthesis

**Scheme 1:**
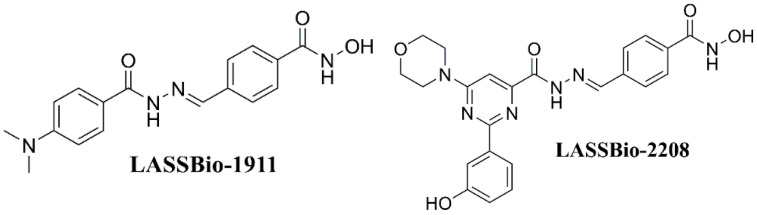
Structures of the compounds used in this work.

LASSBio-1911 and LASSBio-2208 (Scheme 1) were synthesized in the Laboratory of Synthesis and Evaluation of Bioactive Substances (LASSBio, UFRJ, Brazil) following procedures previously described by Rodrigues and colleagues [17]. After confirmation of a purity profile > 95%, the compounds were subjected to in vitro pharmacological assays.

### 4.2. Cell Culture and Treatment

The human prostate cancer cell line PC3 was obtained from the American Type Culture Collection (ATCC^®^ CRL-1435™). The cells were routinely grown in Roswell Park Memorial Institute (RPMI) essential medium (Sigma-Aldrich, St. Louis, MO, USA) containing 10% fetal bovine serum (LGC Biotechnology, São Paulo, Brazil) and 1% penicillin–streptomycin (Sigma Aldrich, St. Louis, MO, USA) in a humidified 5% CO_2_ atmosphere at 37 °C. The cells were cultured to 70–100% confluence. The compounds were dissolved in dimethyl sulfoxide (DMSO) (Sigma-Aldrich, St. Louis, MO, USA) to produce a 10 mM stock solution, and the compounds were dissolved in RPMI at different concentrations. The DMSO concentration did not exceed 0.1% and did not have any effect.

### 4.3. Cell Viability Assay

Cell viability was determined using 3-(4,5-dimethyl-2-thiazyl)-2,5-diphenyl-2H-tetrazolium bromide (MTT) reagent (Sigma-Aldrich, St. Louis, MO, USA) following the method described by Denizot and Lang [44]. Cells were plated at an initial density of 2.5 × 10^4^ cells per well in 96-well plates and incubated for 24 h at 37 °C and 5% CO_2_. After 24 h, the cells were 70–80% confluent and were treated with LASSBio-1911 or LASSBio-2208 at a final concentration of 0.01 to 5 μM and further incubated for 24 or 48 h. The resulting formazan crystals were dissolved in DMSO (100 μL), and the absorbance intensity was measured in a FlexStation 3 microplate reader (Molecular Devices, San Jose, CA, USA) at a wavelength of 570 nm.

### 4.4. Cell Cycle Analysis

The cell cycle distribution was assessed using a BD Biosciences flow cytometry kit according to the manufacturer’s protocol (BD Biosciences, San Jose, CA, USA). Cells were seeded at 2.5 × 10^5^ cells in 12-well plates and incubated with LASSBio-1911 or LASSBio-2208 at a final concentration of 0.1, 1, or 5 μM at 37 °C for 24 h in a humidified chamber containing 5% CO_2_. A total of 10^4^ events of sample cells were recorded by flow cytometry (BD Accuri™ C6 Plus), and the data were analyzed by BD CFlow Plus software (BD Biosciences, San Jose, CA, USA).

### 4.5. Apoptosis Assay

An apoptosis assay was carried out with an Annexin V kit (BD Biosciences, San Jose, CA, USA) by staining the cells according to the manufacturer’s protocol. Briefly, cells (2.5 × 10^5^ cells) were seeded into 12-well plates and incubated with LASSBio-1911 or LASSBio-2208 at a concentration of 0.1, 1, or 5 μM for 24 h, harvested, washed with phosphate-buffered saline (PBS), and resuspended in the indicated binding buffer. Subsequently, Annexin V/FITC and propidium iodide (PI) were added to the cell suspension and incubated for 15 min in the dark at room temperature. After incubation, the samples were analyzed by flow cytometry (BD Accuri™ C6 Plus, BD Biosciences, San Jose, CA, USA), and the data were analyzed by BD CFlow Plus software (BD Biosciences, San Jose, CA, USA).

### 4.6. Cell Scratch Assay

Cellular migration was evaluated by scratch assay according to our previous studies [45,46]. Cells were cultured in 12-well culture plates for 24 h and to 90–100% confluence. The cells were incubated for 24 h with LASSBio-1911 or LASSBio-2208 at final concentrations of 0.01–5 µM. All cell-based scratch assays were performed in the presence of the anti-mitotic reagent cytosine arabinoside (AraC; Sigma-Aldrich, St. Louis, MO, USA) at a final concentration of 10^–5^ M (to inhibit cell proliferation). After treatment with the compounds, the wound areas were observed, and images were acquired with an Evos M5000 (Invitrogen, Thermo Fischer Scientific Inc., Waltham, MA, EUA). The filled area was quantified using the Fiji software (ImageJ, National Institutes of Health, Bethesda, MD, USA).

### 4.7. Western Blot Analysis

The protocol was similar to that described by Oliveira et al. [47] with modifications. The protein content of the cell lysate was determined by a bicinchoninic acid (BCA) protein assay reagent kit (Thermo Fisher Scientific Inc., Waltham, MA, EUA) according to the manufacturer’s protocol. For the Western blot analysis, protein samples (20 μg of protein) were mixed with Laemmli buffer (Bio-Rad Laboratories, Hercules, CA, USA) and subjected to SDS-PAGE (10%). The proteins were transferred onto a nitrocellulose membrane (Amersham Hybond-C extra; GE Healthcare, Chicago, IL, USA) and stained with Ponceau S to assess the efficacy of the transfer. The membranes were incubated with primary antibody overnight with agitation at 4 °C and subsequently with secondary rabbit horseradish peroxidase antibodies (Cell Signaling Technology, Danvers, MA, EUA) for 1 h at room temperature. The following primary and secondary antibody combinations were used for the respective proteins: anti-Caspase 3, anti-JNK, anti-phospho-JNK, anti-JAK2, anti-STAT3, and anti-phospho-STAT3. These antibodies and HRP-conjugated anti-rabbit IgG were obtained from Cell Signaling Technology (Danvers, MA, USA), and anti-β-actin was purchased from Sigma-Aldrich (St. Louis, MO, USA).

### 4.8. IL-6 Quantification

The quantification of IL-6 was performed in the cell culture supernatant. A specific ELISA kit (B&D ELISA OptEIA™, BD Biosciences, San Jose, CA, USA) was used according to the manufacturer’s recommendations. Conditioned medium was collected from untreated cultures or 24 h after treatment with LASSBio-1911 or LASSBio-2208 at concentrations from 0.001 to 5 µM and stimulation with LPS (1 µg/mL). The absorbance was read in a FlexStation 3 microplate reader (Molecular Devices, San Jose, CA, USA) at 450 nm.

### 4.9. Statistical Analysis

All the values are represented as the mean ± standard error of three independent experiments. Statistical analyses were performed with GraphPad Prism 8.02 (GraphPad Software Inc., San Diego, CA, USA) using one-way ANOVA with Dunnett´s post-test, and statistical significance was defined as * *p* < 0.05.

## 5. Conclusions

LASSBio-1911 and LASSBio-2208 were rationally designed and synthesized by leveraging their HDAC6/8 inhibitory functionality and dual inhibitory functionality against HDAC6 and PI3K, respectively. In this study, we established LASSBio-1911, a potent HDAC6/8 inhibitor, and LASSBio-2208, a potent dual PI3K/HDAC6 inhibitor, as two novel therapeutic potential drugs against prostate cancer with high metastatic potential and limited treatment options.

**Figure 8:**
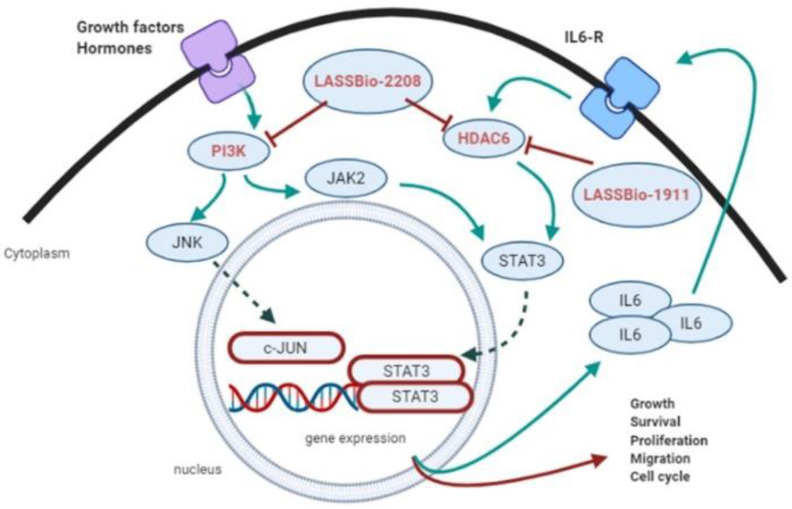
Mechanism of action of the LASSBio-1911 and LASSBio-2208 compounds. Created in BioRender.com (accessed on 15 February 2021).

We showed, for the first time, the antitumor activity and the underlying molecular mechanisms of LASSBio-1911 and LASSBio-2208 in prostate cancer. Our results show that LASSBio-1911 and LASSBio-2208 can induce G2/M cell cycle arrest and reduce cell viability by inducing apoptosis and inhibiting the JNK pathway. Additionally, the inhibitors were able to reduce cell migration, contributing to a reduction in metastasis through the inhibition of the JAK2/STAT3 signaling pathway and the inhibition of IL-6 secretion (Figure 8).

In conclusion, the results described herein reinforce the importance of developing novel multitarget drug candidates structurally designed by combining the pharmacophoric subunits necessary for molecular recognition by the selected targets, in this case, HDAC and PI3K isoforms, to amplify the therapeutic benefits in the treatment of prostate cancer, a multifactorial disease.

## Figures and Tables

**Table 1:** Effects of LASSBio-1911 and LASSBio-2208 on the cell cycle phase distribution of PC3 cells.

Cell Cycle Phases (%)	Concentration	G0/G1	S	G2 + M
Control	-	13.2 ± 2.49	43.8 ± 5.57	31.09 ±9.0
Vehicle	-	12.6 ± 2.8	38.6 ± 2.8	49.6 ± 9.7
LASSBio-1911	0.1 µM	2.86 ± 0.8	3.2 ± 1.1	82.1 ± 3.9 *
	1 µM	3.46 ± 0.9	3.1 ± 0.8	81.8 ± 4.0 *
	5 µM	2.3 ± 0.7	2.9 ± 0.87	82.1 ± 4.8 *
LASSBio-2208	0.1 µM	3.2 ± 0.1	3.4 ± 0.3	81.0 ± 2.4 *
	1 µM	2.9 ± 0.75	2.9 ± 0.8	81.0 ± 5.5 *
	5 µM	2.63 ± 0.2	3.9 ± 0.7	81.0 ± 4.0 *

## Data Availability

The data are available within the article or from the corresponding author on reasonable request.
